# Enhanced Interstitial Fluid Extraction and Rapid Analysis via Vacuum Tube‐Integrated Microneedle Array Device

**DOI:** 10.1002/advs.202308716

**Published:** 2024-03-19

**Authors:** Yuanting Xie, Jinhua He, Wenqing He, Tayyaba Iftikhar, Chuangjie Zhang, Lei Su, Xueji Zhang

**Affiliations:** ^1^ Guangdong Key Laboratory for Biomedical Measurements and Ultrasound Imaging National‐Regional Key Technology Engineering Laboratory for Medical Ultrasound School of Biomedical Engineering Shenzhen University Medical School Shenzhen 518060 China; ^2^ Shenzhen Key Laboratory of Nano‐Biosensing Technology Marshall Laboratory of Biomedical Engineering International Health Science Innovation Center Shenzhen University Medical School Shenzhen University Shenzhen 518060 China; ^3^ Guangdong Laboratory of Artificial Intelligence and Digital Economy (SZ) Shenzhen University Shenzhen 518060 China

**Keywords:** biomarkers, biosensors, interstitial fluid, ISF extraction, microneedle, POCT

## Abstract

Advancing the development of point‐of‐care testing (POCT) sensors that utilize interstitial fluid (ISF) presents considerable obstacles in terms of rapid sampling and analysis. Herein, an innovative strategy is introduced that involves the use of a 3D‐printed, hollow microneedle array patch (MAP), in tandem with a vacuum tube (VT) connected through a hose, to improve ISF extraction efficiency and facilitate expedited analysis. The employment of negative pressure by the VT allows the MAP device to effectively gather ≈18 µL of ISF from the dermis of a live rabbit ear within a concise period of 5 min. This methodology enables the immediate and minimally invasive measurement of glucose levels within the body, employing personal healthcare meters for quantification. The fusion of the VT and MAP technologies provides for their effortless integration into a comprehensive and mobile system for ISF analysis, accomplished by preloading the hose with custom sensing papers designed to detect specific analytes. Moreover, the design and functionality of this integrated VT‐MAP system are intuitively user‐friendly, eliminating the requirement for specialized medical expertise. This feature enhances its potential to make a significant impact on the field of decentralized personal healthcare.

## Graphical abstract

A 3D‐printed hollow MAP complemented with a hose‐linked vacuum tube (VT) is developed for efficient and rapid ISF collection and analysis. The visual representation showcases the MAP's gentle skin penetration, enhanced ISF extraction, and sensing functions. ISF analysis versatility with custom sensing papers and prioritize user‐friendly healthcare advancement, eliminating specialized training is emphasized.

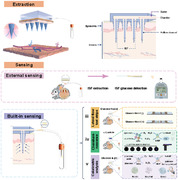

## 1 Introduction

Societal aging and the development of telehealth have increased the demand for rapid health monitoring at home. Therefore, there is considerable emphasis on POCT devices or systems designed for on‐site, easy‐to‐operate, rapid detection of health conditions, including the diagnosis of sudden diseases.^[^ ^1^, ^2^, ^3^, ^4^ ^]^ Among various body fluids for POCT, blood is currently the most common choice, e.g., the widely used home blood glucometer. However, the inevitable invasive nature of blood sampling during POCT often causes patient discomfort, blood clotting, infections, and other related issues.^[^ ^5^, ^6^ ^]^ In this regard, dermal ISF, formed by blood transcapillary filtration, has emerged as a significant source of technological innovation for advancing POCT sensors. ISF allows for pain‐free and infectious risk‐free collection, and the bio‐analytes it contains exhibit a high correlation with blood components.^[^ ^7^, ^8^ ^]^ The liquid content in the dermis approximately accounts for 70% including 30% of bound water and 40% of free ISF. This can be roughly estimated that 120 and 24 µL cm^−2^ of ISF could be available in the thickest (3 mm for palms, back, and soles) and thinnest regions (0.6 mm for eyelids) of the dermis, respectively.^[^ ^9^, ^10^, ^11^ ^]^ Moreover, due to the presence of hydrophilic extracellular matrix (ECM) components (such as collagen, elastic fibers, and extrafibrillar matrixes), ISF exhibits a hydrogel‐like viscosity.^[^ ^9^, ^12^ ^]^ These tissue characteristics of the dermis pose inherent challenges in collecting ISF. Conventional methods to extract dermal ISF, including the wick method,^[^ ^13^ ^]^ suction blisters,^[^ ^14^ ^]^ micro‐dialysis,^[^ ^15^ ^]^ and reverse iontophoresis,^[^ ^16^ ^]^ typically have significant drawbacks. For instance, the wick method is invasive and requires post‐processing procedures to collect the ISF from the wick, suction blisters require the use of high pressure, leading to local damage and inflammatory response, and distortion of analyte concentrations, micro‐dialysis is invasive and causes the 5 to 10‐fold dilution of analytes, and reverse iontophoresis also leads to severe dilutions of analytes.^[^ ^9^, ^17^ ^]^ Therefore, there is a high demand for new methods that can efficiently extract dermis ISF.

MAPs are arrays of micrometer‐sized needles that are capable of penetrating the skin into the dermis but without reaching the blood vessels and pain receptors in the deeper layer of the dermis, making them discomfort‐free and pain‐free platforms for ISF extraction.^[^ ^17^, ^18^, ^19^ ^]^ MAPs as an ISF extraction platform open up new opportunities for integrating highly sensitive and selective biosensing methodologies to create all‐in‐one devices for on‐site, facile, comprehensive analysis of ISF, which is favorable for POCT applications.^[^ ^19^, ^20^, ^21^, ^22^, ^23^, ^24^, ^25^, ^26^ ^]^ However, the ISF extraction amounts of most current MAPs were only 1–10 µL at the cost of minutes to tens of minutes,^[^ ^11^, ^17^, ^27^ ^]^ which is inadequate and not timely enough for many rapid POCT sensing applications. The minuscule ISF extraction amount is significantly lower than the estimated value mentioned above. The inefficiency of ISF extraction has led to a challenging situation where many existing in vitro diagnostic methodologies require reevaluation to determine their suitability for rapid and comprehensive ISF analysis at point‐of‐care applications when integrated with MAPs. There is a high demand for new MAP‐based ISF POCT sensors. On the other hand, hydrogel‐based swellable MAPs used in the POCT applications often require ISF collection from the water‐locking gel, necessitating additional post‐processing procedures. Other MAP‐based ISF extraction methods, although excel in large‐volume in vivo ISF collection, such as high pressure‐assisted capillary force‐driving ISF extraction^[^ ^28^ ^]^ and electroosmosis‐driving ISF extraction,^[^ ^29^ ^]^ employed high vacuum pressures generally in the range of tens of kPa. These pressures can damage the skin causing bruising and blisters, and requiring extra specialized pressure‐generating instruments and accessories or sophisticated operation procedures, which are unsuitable for POCT applications.

**Figure 1:**
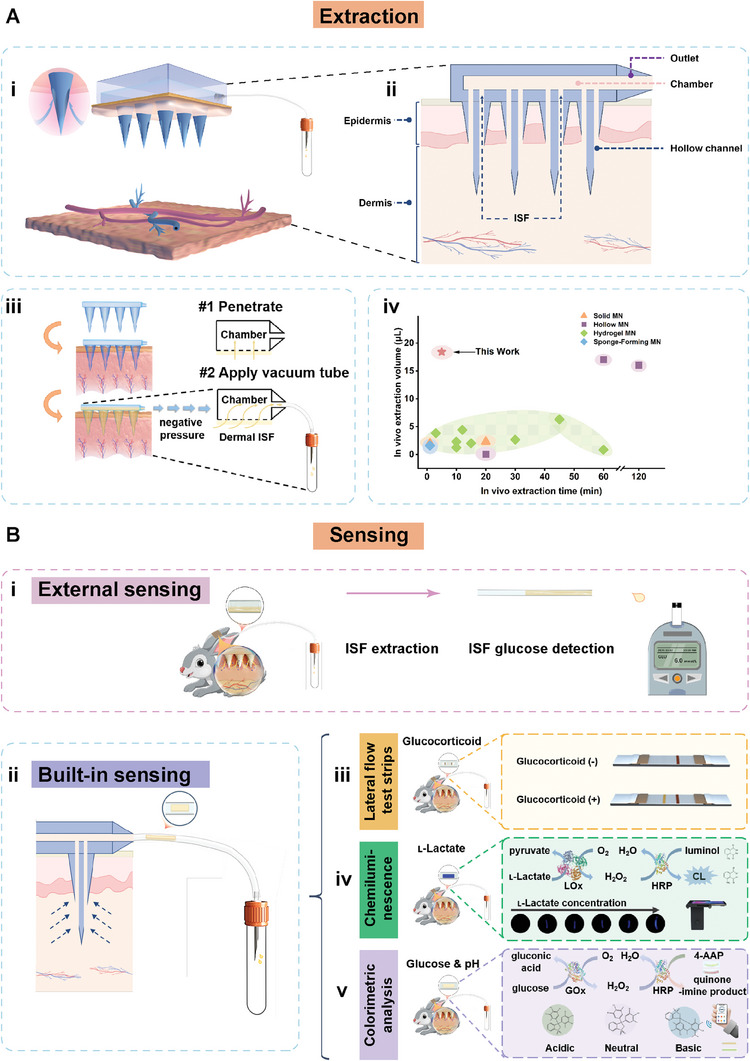
Schematic illustration of the VT‐integrated MAP bifunctional device for rapid extraction and analysis of ISF. A) The VT‐integrated MAP device for extraction of ISF. Structure illustration of the VT‐integrated MAP device i). Cross‐section of the MAP inserted into the dermis ii). Illustration of the extraction of ISF with the VT‐integrated MAP device iii): The penetration of the MAP into the skin to reach the dermal zone (#1). The flow of ISF from the dermal layer into the internal chamber of the patch and then into the hose under the negative pressure driving force (#2). In vivo ISF extraction amount versus extraction time with data from this study and previous reports^[^ ^11^, ^28^, ^30^, ^31^, ^32^, ^33^, ^34^, ^35^, ^36^, ^37^, ^38^, ^39^, ^40^ ^]^ iv). B) The VT‐integrated MAP device for analysis of ISF. Direct detection of ISF glucose by transferring the extracted ISF from the hose to a commercial glucometer i). Construction of a versatile sensing platform by prefilling the hose with the analyte‐responsive sensing units ii), including LFTS iii), chemiluminescent iv), and colorimetric sensing papers v) for in vivo ISF analysis.

To tackle these challenges, we present a 3D‐printed hollow MAP combined with a hose‐linked vacuum tube (VT) to improve ISF extraction and subsequent ISF analysis (**Figure** **1**). With it, we could collect 18.42 ± 1.02 µL ISF within 5 min from a live New Zealand (NZ) rabbit ear skin. This corresponds to a mean extraction rate of 0.0368 µL min^−1^ per needle, which is currently the highest efficiency in MAP‐based minimally invasive in vivo sampling, to the best of our knowledge. In addition, the volume of the free ISF rapidly flowing into the hose reached as high as 14.80 ± 3.16 µL, enabling simple, direct, and quantitative in vitro analysis of ISF glucose by dropping the ISF from the hose onto a commercial glucometer. It is also of great significance that the VT‐integrated MAP device can serve as a platform to construct versatile sensing systems by pre‐filling the hose with the analyte‐responsive sensing units, including lateral flow test strip (LFTS), chemiluminescent and colorimetric sensing papers. As such, the extracted ISF can be instantly analyzed in the hose by employing customized sensing units for specific requirements. In addition, the sampling and sensing operations are easily accessible and user‐friendly, eliminating the need for trained medical professionals.

## 2 Results and Discussion

### 2.1 Design and Characterizations of the MAP of the VT‐Integrated MAP Device

**Figure 2:**
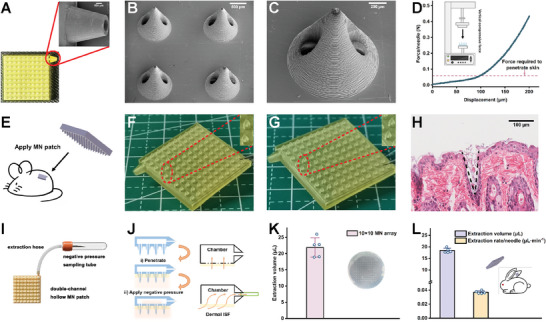
Characterizations of the MAP and evaluation of ISF extraction performance of the VT‐integrated MAP device. A) The photos of a 10 × 10 array on the 21 × 21 mm plate. B,C) SEM images of a bi‐channel hollow MAP (B) and a single bi‐channel hollow microneedle (C), respectively. D) Compression force versus displacement curve. Inset: schematic representation of the MAP compression tests. E) Schematic representation of the MAP applied in a mouse model. F, G) Optical micrographs of the 10 × 10 MAP before and after penetration, respectively. H) Hematoxylin & eosin (H&E)‐staining of the mouse skin pierced by the MAP. The black dashed line indicates the insertion wound. Scale bar: 100 µm. I) Schematic representation of the MAP‐based extraction device. J) Illustration of the extraction of ISF with the VT‐integrated MAP‐based device: Penetration: the MAPs pierced into the skin to reach the dermal zone i). Negative pressure: the ISF flows from the dermal layer into the internal chamber of the patch and then into the hose under the negative pressure driving force ii). K) The extraction performance of the MAP‐based device within 5 min using agarose gel as skin model (*n* = 5). Inset: the photo of the agarose gel after removal of the MAP after a 5‐min application period. L) In vivo ISF extraction performance of the MAP‐based device in the live NZ rabbit ear skin (*n* = 5), including the ISF extraction volume (blue column) and ISF extraction rate (orange column).

As schematically illustrated in Figure 1A, and Figure S1 (Supporting Information), the MAP device consists of a patch body with an internal chamber, a bi‐channel hollow microneedle array at the base, and an outlet port on one side. The outlet port of the MAP connects to a hose, and the opposite end of the hose connects to a VT capable of supplying the negative pressure driving force. This configuration transforms the entire device into a hand pump, enabling rapid extraction of ISF from the skin. We fabricated the MAP using biocompatible polymethylacrylate (PMA) resins on a micro‐nano 3D printer, and the fabrication process is schematically illustrated in Figure S2 (Supporting Information). **Figure** **2**A and Figure S3 (Supporting Information) show the photos of the 10 × 10 MAP. As shown, the patch had a thin 21 × 21 mm square shape, and the microneedles at the base of the MAP body exhibited a tip height of 1,000 µm to ensure a pain‐less insertion into and sufficient ISF extraction from the epidermal layer of the skin.^[^ ^9^, ^12^ ^]^ Figure 2B,C show SEM images of the MAP and a single microneedle tip, respectively. The microneedles had a conical morphology supporting a secure and effective penetration.^[^ ^41^ ^]^ The needle interval between adjacent tips, i.e., center‐to‐center distance, was optimized to 2,000 µm since the higher density of the needles may decrease the insertion probability.^[^ ^12^, ^37^ ^]^ The conical microneedle tip had two hollow channels of a 250‐µm diameter. The mechanical strength of the MAP was evaluated by compression tests. Figure 2D shows the curve obtained for compression force versus displacement. The single microneedle could withstand the loading force higher than 0.058 N needle^−1^, which is the minimal force required for puncture of human skin without microneedle breakage.^[^ ^42^ ^]^ The yield strength of the MAP was calculated to be 22.77 MPa (Figure S4, Supporting Information), indicating that the MAP possesses sufficient mechanical strength for skin penetration.^[^ ^43^ ^]^

Furthermore, we assessed the in vivo skin penetration capability of the MAP using BALB/c mice (Figure 2E). Our observations revealed that the MAP readily penetrated the dorsal skin of the mice with just a gentle thumb press. As demonstrated in Figure 2F,G, the MAPs after insertion into the mice's skin showed negligible morphology change compared to the original MAPs, suggesting a good deformation resistance, thereby reducing the risk of microneedle breakage during the penetration of the skin. The histological results, as shown in Figure 2H, revealed that the tip of the microneedle could pierce the epidermis to reach the dermis but have no access to the capillaries and nerve endings, thereby enabling the extraction of the dermal ISF painlessly. Moreover, we observed that the skin, once pierced by the MAPs, demonstrated the ability to reseal shortly after removing the MAPs. As shown in Figure S5 (Supporting Information), the microneedle‐induced micropores disappeared gradually in 30 min after removing the MAPs, indicating that the penetration was minimally invasive for the skin, minimizing the risk of microbe infiltration‐induced infections.

### 2.2 Evaluation of Extraction Performance of the VT‐Integrated MAP Device Using in Vitro Skin Model and Live NZ Rabbit

In vitro models as tissue surrogates could obviate the issue of sample heterogeneity.^[^ ^44^ ^]^ The ISF extraction performance of the VT‐integrated MAP device was first assessed using the widely accepted agarose gel skin model.^[^ ^12^ ^]^ After pressing the MAP into the agarose gel (1.5 wt.%), the VT was connected to offer the negative pressure to extract the liquid from the gel (Figure 2I,J; Video S1, Supporting Information). As shown in Figure 2K, within 5 min, the extracted liquid volume reached 21.88 ± 2.97 µL, faster than the extraction rates of most of the MAPs on the agarose gel skin model.^[^ ^34^, ^45^, ^46^ ^]^ After removing the MAP, the rubbing of the MAP can be clearly seen on the agarose gel (Figure 2K, right). We studied the effect of the microneedle array quantity on the extraction performance of the MAP‐based device by using MAPs with different array numbers (5 × 5, 8 × 8, 9 × 9, 10 × 10, respectively). As shown in Figure S6 (Supporting Information), the extracted ISF volume raised from 12.08 ± 0.78 µL to 21.88 ± 2.97 µL with the increase in the array number from 5 × 5 to 10 × 10.

Conversely, we examined the impact of negative pressure on the extraction performance of the MAP‐based device by employing a vacuum pump equipped with a standard vacuum gauge (0–20 kPa) to generate a large negative pressure. As anticipated, under the kPa‐level negative pressure generated by the vacuum pump the extraction amount increased. However, the gel surfaces were also seriously damaged (Figure S7, Supporting Information). Indeed, significant negative pressures have the potential to cause skin damage, as illustrated in Figure S8 (Supporting Information), consistent with previous reports.^[^ ^28^, ^47^ ^]^ In contrast, the VT demonstrated the ability to deliver a modest yet sufficient negative pressure (75 Pa, calculated according to the Chinse National standard of the VT: WS/T 224–2018). This allowed our lab‐made MAP‐based device to effectively extract liquid from the model skin along with a negligible invasion.

Subsequently, we investigated the in vivo ISF extraction performance of the MAP‐based device with the live, healthy NZ rabbit ear. Rabbit ear skin is histologically similar to human skin, wherein the thickness of the stratum corneum (SC) of rabbit ear skin is 11.7 ± 0.5 µm thick, closer to human skin (SC, 10–20 µm thick) than mouse skin (SC, 5 µm thick).^[^ ^48^ ^]^ As shown in Figure 2L, within 5 min, the volume of the extracted ISF reached 18.42 ± 1.02 µL. Such an ISF extraction performance is the highest among the current MAP‐based methods, as shown in Figure 1A(iv). With the VT‐integrated MAP device, the in vivo ISF extraction volume reached 18.42 ± 1.02 µL within 5 min, corresponding to an in vivo ISF extraction rate (3.68 µL min^−1^). The in vivo ISF extraction ability of a single hollow microneedle reached 0.184 µL within 5 min, corresponding to an extraction rate of 0.0368 µL min^−1^ per needle, which is so far the highest efficiency in a microneedle‐based minimally invasive in vivo sampling manner (Table S2, Supporting Information). The ISF volume entering the hose was further measured to be 14.80 ± 3.16 µL, which could ensure the simple, direct, and quantitative analysis of the ISF glucose via a commercial glucometer, as below. It is worth noting that with the VT‐integrated MAP device, the ISF can be collected and stored in the hose for subsequent analysis, unlike the hydrogel MAPs that need additional time‐consuming posttreatment procedures to isolate the extracted ISF from the water‐locking hydrogel, showing the simplicity of our technique.

### 2.3 Rapid Analysis of Dermal ISF Glucose in the Live NZ Rabbit Ear with a Commercial Glucometer

As expected, upon transferring the ISF from the hose onto the testing pad of the glucometer, the glucose content could be promptly read out. In this way, the ISF glucose of the live, healthy NZ rabbit after 12‐h fasting was obtained to be 6.0 mM (Figure S9, Supporting Information), aligning with previous reports.^[^ ^49^ ^]^ These results suggest that our device can be combined conveniently with specific commercial personal medical sensors that require only several µL‐level sample volumes, thereby realizing rapid analysis of skin ISF.

In addition, the MAP was made of non‐swelling light‐curing PMA resin, indicating the potential of the reuse of the MAP, which was investigated by using multiple skin insertion tests for ten finger press‐release cycles on the porcine skin due to its histological similarity to human skin. SEM images of the microneedle tips after 10th cycle are shown in Figure S10 (Supporting Information). The microneedle tips preserved their structural integrity. Besides, the MAP also underwent the 10‐time compressive cycle test with a maximum force of 20 N (much larger than the required force for skin penetration^[^ ^11^, ^50^ ^]^). As shown in Figure S11 (Supporting Information), the recorded displacement change was as small as 40 µm, indicating the good mechanical strength of the MAP. Both the SEM characterizations and the compressive studies confirm the recyclable property of the MAP. Moreover, we found that the PMA‐resin MAPs could endure sterilization treatments including 75% alcohol, UV‐light, and autoclaving, guaranteeing the reusable biosafety of the MAPs.

On the other hand, our device has functional scalability. It can serve as a platform for constructing versatile sensing systems by equipping the hose with built‐in sensing units, forming a miniaturized extraction‐sensing bifunctional device, as follows.

### 2.4 Rapid Lateral Flow Analysis of ISF in live NZ Rabbit Ear with the VT‐Integrated MAP‐Based Device

**Figure 3:**
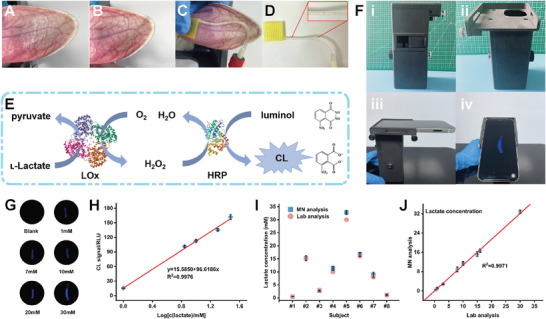
The VT‐integrated MAP device for rapid extraction, lateral flow analysis, and chemiluminescent analysis of dermal ISF. A, B) Photos of a live NZ rabbit ear topically treated with GC (A) and after absorption of GC (B), respectively, as indicated by the red frame zone. C) The application of MAP patch adjacent to the GC‐applied zone. D) Photo of the LFTS in the hose of our VT‐integrated MAP device after detecting interstitial GC in the live rabbit ear. E) The principle of chemiluminescent assay of lactate. F) Views of the lab‐made dark box. G) The CL intensity changes of the hoses in response to the different lactate concentrations from 0, 1, 7, 10, 20, and 30 mM, respectively. H) The CL intensity of the hoses in response to the different lactate values from 1.0, 7.0, 10.0, 20.0, and 30.0 mM, respectively (*n* = 3). I, J) Lactate concentrations of the agarose gel skin model measured by the lab‐made VT‐integrated MAP device (I) and their correlations to standard values (J) (*n* = 3).

LFTS sensors are among the most popular POCT devices for rapid diagnosis.^[^ ^51^ ^]^ However, they need a relatively large volume of samples, usually more than 30 µL,^[^ ^52^ ^]^ making them an onlooker during point‐of‐care ISF analysis due to the low ISF extraction amount and slow ISF extraction rate with the current microneedle technologies. Due to the improvement of in vivo ISF extraction performance using our MAP‐based device, ISF in vivo can be rapidly analyzed using a commercially available LFTS for the first time by incorporating it into our MAP‐based device. In dermatology, topical glucocorticoid (GC) treatments are widely used for anti‐dermatitis. For instance, dexamethasone as a synthetic GC can treat eczema.^[^ ^53^ ^]^ Nevertheless, certain cosmetics companies engage in illegal practices by incorporating GC into their facial mask and cream products to achieve immediate benefits, such as rapid brightening and anti‐aging effects on the skin. The long‐term use of GC can result in troublesome side effects such as dependence, facial erythema, etc. ^[^ ^54^ ^]^ Therefore, rapid detection of GC in the skin is of great significance. To this end, a commercially available GC‐detecting LFTS as a model was cut into a 1.5 × 20 mm and then inserted into the hose of our device, forming an integrated bifunctional device. Dexamethasone cream was dabbed onto the rabbit ear skin and allowed to be fully absorbed (**Figure** **3**A,B). Then, the MAP device was pressed onto the rabbit ear adjacent to the cream‐applied area (Figure 3C; Figure S12, Supporting Information). After connecting the VT to initiate ISF extraction, both the test and control lines in the hose became visible within 5 min (Figure 3D). The positive results obtained for GC indicate that the VT‐integrated MAP device, cleverly combining a commercially available LFTS, can rapidly detect the bio‐analytes in the skin.

### 2.5 Rapid Chemiluminescent Analysis of ISF in Live NZ Rabbit Ear with the VT‐Integrated MAP Device

Chemiluminescence (CL) has performed well in current bioanalysis applications because of its high sensitivity and low background interference. In addition, the lack of need for external light sources also makes CL attractive for miniaturized POCT devices.^[^ ^55^ ^]^ Herein, CL has been applied for rapid analysis of in vivo ISF for the first time by incorporating it into our MAP‐based device. To achieve this objective, a nitrocellulose paper fragment (1.5 × 10 mm), precoated with the luminol‐lactate oxidase‐horseradish peroxidase (HRP) chemiluminescent agents, was inserted into the fore‐end of the hose close proximately to the patch's outlet port. In the presence of lactate in the hose, the bi‐enzyme system's catalysis initiated CL of luminol (Figure 3E).^[^ ^56^ ^]^ The CL could be observed in a custom‐designed light‐tight box (Figure 3F; Figure S13, Supporting Information) using a smartphone, allowing for the quantification of the lactate levels. The box, manufactured through cost‐effective 3D printing technology with black acrylonitrile‐butadiene‐styrene (ABS) polymers, featured a sliding gate for sample ingress and egress, a liftable plate with a sample groove for focusing, and a small window for a smartphone image capture (Video S2, Supporting Information). As shown in Figure 3G, the hoses exhibited different CL intensities in response to the lactate concentrations ranging from 0.5–30 mM. Figure 3H depicts the corresponding calibration curve, showing an outstanding linear relationship with the limit of detection (LOD) of 56.7 µM, which could be used to indicate the average human lactate level and hyperlactatemia as well as lactic acidosis.^[^ ^57^ ^]^ The detection performance of the CL test paper‐loaded hose was validated using a commercial CL analysis instrument with a set of solutions containing eight different lactate concentrations as blind samples. Figure 3I,J shows the obtained lactate concentration. As revealed, the detected lactate concentration with our device showed excellent agreement with those measured by the CL analysis instrument. These results demonstrate our device's practical suitability for reliable lactate analysis.

The extracted ISF could be analyzed rapidly with the MAP‐based device integrated with the CL sensing hose. After connecting the VT to initiate rapid ISF extraction, the sensing unit of the hose emitted light upon the entry of ISF. After immediately transferring the hose into the lab‐made dark box for imaging, the CL intensity change was captured by a smartphone to give the live NZ rabbit ear skin ISF lactate concentration of 10.25 mM.^[^ ^58^ ^]^ The whole ISF extraction and analysis were completed within 3 min.

### 2.6 Rapid Extraction and Colorimetric Analysis of ISF in Live NZ Rabbit Ear with the VT‐Integrated MAP Device

**Figure 4:**
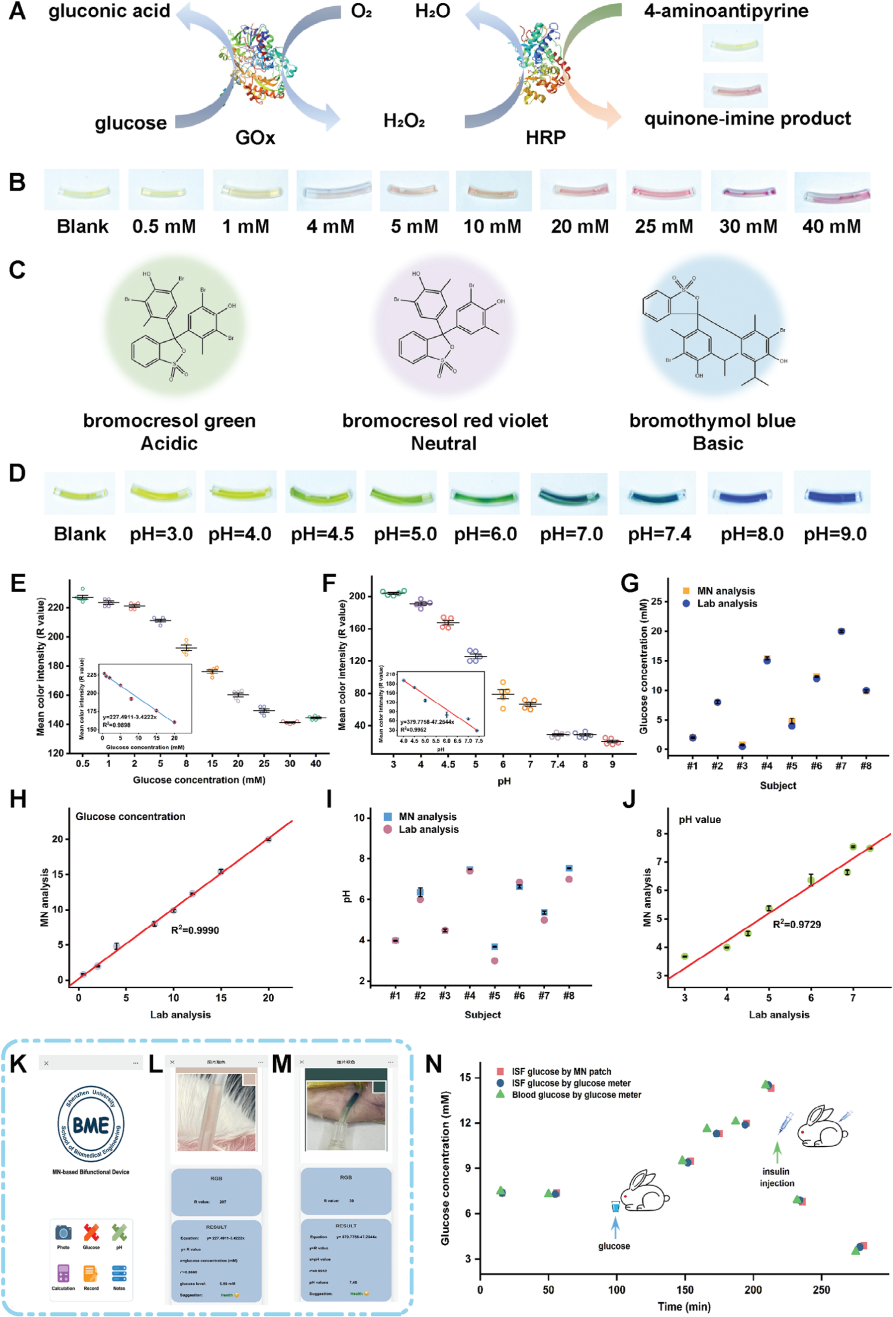
The VT‐integrated MAP device for rapid extraction and colorimetric analysis of ISF. A) The principle of glucose colorimetric assay of the VT‐integrated MAP device. B) The color of the hoses in response to the different glucose concentrations from 0, 0.5, 1, 4, 5, 10, 20, 25, 30, and 40 mM, respectively. C) The principle of pH colorimetric assay of the MAP‐based device. D) The color of the hoses in response to the different pH values from 3.0, 4.0, 4.5, 5.0, 6.0, 7.0, 7.4, 8.0, and 9.0, respectively. E) The R‐value versus glucose concentration (*n* = 5). Inset: the linear relationship of the R‐value versus the glucose concentration in a range of 0–20 mM. F) The R‐value versus pH value (*n* = 5). Inset: the linear relationship of the R‐value versus pH value (4.0–7.4). G, H) Glucose concentrations of the agarose gel skin model measured by the VT‐integrated MAP device (G) and their correlations to standard values (H) (*n* = 3). I, J) pH values of the agarose gel skin model measured by the VT‐integrated MAP device (I) and their correlations to standard values (J) (*n* = 3). K) The colorimetric analysis APP on a smartphone for glucose and pH analysis. L) The in vivo glucose concentration detection of the rabbit skin via the VT‐integrated MAP device and result readout via the smartphone app. M) The in vivo pH detection of the rabbit skin via the VT‐integrated MAP device and the result readout via the smartphone app. N) Variation in interstitial glucose concentrations detected with MAP (red cube) and glucometer (purple dot) and in vein blood glucose concentrations measured with a commercial glucometer (green triangle).

To endow the VT‐integrated MAP device with colorimetric analysis capabilities, we inserted a small piece of dry nitrocellulose paper (1.5 × 10 mm) pre‐decorated with colorimetric agents into the fore‐end of the hose, near the outlet port of the patch. It has been known that ISF pH is lower in diabetes mellitus, which is also one of the causes of insulin resistance.^[^ ^59^ ^]^ Therefore, it is significant to develop methods for detecting both ISF pH and glucose. For glucose sensing, the colorimetric agents consisted of 4‐aminoantipyrine (4‐AAP) and a bi‐enzyme system including glucose oxidase (GOx) and HRP. In the presence of glucose in the hose, the catalysis of the bi‐enzyme system led to the oxidation of 4‐AAP to produce chromogenic quinone‐imine product (**Figure** **4**A),^[^ ^60^ ^]^ causing the quick color change in the hose from light yellow to burgundy red within 5 min. As shown in Figure 4B, the hoses exhibited different colors in response to varied glucose concentrations ranging from 0.5–40 mM. For sensing pH, the mixture of bromocresol green, bromocresol red violet, and bromothymol blue was introduced into the hose as the colorimetric indicator (Figure 4C). Based on it, the pH value of the solution in the hose could be visualized by the color change of the hose. As shown in Figure 4D, the hoses exhibited different colors in a rapid response within 2 min to pH values from 3.0 to 9.0. The smartphone could also capture these color changes. To minimize the interferences of the stray light, the digital photos were acquired under the same lighting conditions. The RGB values of the captured image colors can be analyzed to depict the analyte's concentration quantitatively. As revealed by Figure S14A (Supporting Information), the R, G, and B values decreased with increasing glucose concentration. The R‐value versus glucose concentration shows a robust linear region in the range of 0–20 mM at a high linear correlation coefficient R^2 ^= 0.9898 with the LOD of 0.51 mM (Figure 4E). Figure S14B (Supporting Information) shows the RGB changes in the colorimetric detection of pH. The R‐value indicates a robust linear region in the pH 4.0–7.4 range at a high linear correlation coefficient R^2 ^= 0.9952 (Figure 4F). Then, the integrated bi‐functions of our device were first tested using the agarose gel skin model. A series of solutions containing eight different glucose concentrations and eight pH values, respectively, were used to prepare a batch of 1.5 wt.% agarose gels as blind samples. The obtained colorimetric assay results were verified with the commercial glucometer and a standard pH meter. Figure 4G,H shows the obtained glucose concentration of the liquid extracted from the agarose gels. Figure 4I,J shows the obtained pH values of the liquid extracted from the agarose gels. As revealed, the detected glucose concentration and pH values with our device showed excellent agreement with conventional instrumental analysis. These results effectively demonstrate the practical suitability of our MAP‐based device for extraction and reliable analysis of ISF.

Before in vivo analysis, the extraction of the R values from the color changes of the sensing unit of the hose and the transformation of the R values to the analyte concentrations were integrated into a lab‐designed smartphone app (Figure 4K). During in vivo ISF extraction and analysis, upon connecting the VT to start the extraction, the sensing unit of the hose underwent a glucose‐responsive color change, transitioning from light yellow to light red within 3 min. The color change was captured and processed on a smartphone to give the rabbit ear skin ISF's glucose concentration of 5.99 mM (Figure 4L). The glucose concentration in the rabbit ear vein blood was also measured using a commercial glucometer to be 6.00 mM. The glucose level detected with our MAP‐based device agreed well with that measured with the glucometer. As for sensing in vivo ISF pH, the MAP patch was reused after being immersed in 75% alcohol and sterilized with saline several times. Following this, a new hose pre‐filled with the colorimetric pH sensing unit was introduced. After connecting the VT to start the extraction, the color of the sensing unit of the hose changed from yellow to deep blue within 2 min. Accordingly, the color change was captured and processed on a smartphone to give the pH value of 7.4 (Figure 4M), consistent with that in the rabbit ear vein blood measured using a standard pH meter.

Furthermore, the VT‐integrated MAP bifunctional device was applied to monitor the variations in interstitial glucose concentrations caused by oral glucose intake and insulin intramuscular injection, which was verified by concomitantly measuring the variations of vein blood glucose concentrations with a commercial glucometer. Oral glucose intake can cause an increase in glucose concentrations of body fluids and insulin injection will reverse this trend. As shown in Figure 4N, the interstitial glucose concentration obtained with our VT‐integrated MAP device was qualitatively and quantitatively consistent with that measured with the commercial glucometer. Additionally, the detected interstitial glucose concentrations were consistent with the vein blood glucose concentrations. These observations, on the one hand, suggest that the interstitial glucose concentrations followed the vein blood glucose concentrations all along. On the other hand, confirms the reusability of the MAP and the reliability of the sensing units of our VT‐integrated MAP device.

## 3 Conclusion

To summarize, we have presented a groundbreaking solution for ISF rapid extraction and analysis via a 3D‐printed hollow solid MAP complemented by a hose‐linked VT. Leveraging the negative pressure driving force as low as 75 Pa offered by the VT, the MAP device efficiently collected 18.42 ± 1.02 µL of ISF within a 5‐min timeframe, corresponding to a high in vivo ISF extraction rate of a single hollow microneedle (0.0368 µL min^−1^ per needle). Both the volume and rate of in vivo ISF extraction exceed those achieved by state‐of‐the‐art hydrogel MAPs. Additionally, with the VT‐integrated MAP device, ISF can be easily collected and stored in the hose for subsequent analysis. Our device is miniaturized, portable, and user‐friendly, making it highly suitable for POCT applications.

Moreover, the quantity of in vivo ISF extraction ensures straightforward, direct, and quantitative analysis of the ISF glucose by simply transferring the ISF from the hose onto a commercial glucometer. This device offers ease of use for home healthcare monitoring, eliminating the need for trained healthcare professionals to operate it. This accessibility enhances the availability of diagnostic information, providing a superior alternative to more painful and inconvenient fingertip blood collection methods. Therefore, we firmly believe that this technique disrupts the currently predominant practice of fingertip blood collection for in‐body glucose self‐monitoring using glucometers and has the potential to benefit diabetes patients worldwide. Furthermore, it suggests that the VT‐integrated MAP device can provide ISF samples that can be directly utilized for rapid analysis by other commercially available medical sensors requiring small sample volumes.

Importantly, our device exhibits functional scalability and serves as a platform for the construction of versatile point‐of‐care sensing systems by pre‐filling the hose with analyte‐responsive sensing units, including LFTS, chemiluminescent, and colorimetric sensing papers, as demonstrated previously. Based on this, the extracted ISF can be instantaneously analyzed within the hose using customized sensing units tailored to specific requirements, without the need for an external bulk analysis apparatus. We believe in the possibility of incorporating additional methodologies into our device further to develop novel POCT sensors.

## 4 Experimental Section

### Chemicals and Materials

Horseradish peroxidase (HRP), glucose oxidase (GOx, from Aspergillus niger, 180 U mg^−1^), 1 M glucose standard solution, lactate oxidase (LOx, 20 U mg^−1^), ʟ‐lactic acid, and agarose powder were purchased from Aladdin Chemistry Co. Ltd. (China). Sodium bicarbonate (NaHCO_3_), sodium carbonate (Na_2_CO_3_), hydrochloric acid (HCl), sodium hydroxide (NaOH), rhodamine 6G, 4‐aminoantipyrine (4‐AAP), chitosan powder (medium molecular weight), acetic acid, pH standard solutions (pH 4.0, pH 6.86, pH 9.0), bromocresol red violet, bromocresol green, and bromothymol blue were obtained from Macklin Biochemical Co., Ltd., China. Phosphate buffer solution (PBS) (1.0 m, pH 7.4), 4.0% paraformaldehyde, and nitrocellulose paper were purchased from Beyotime Biotechnology Co., Ltd., China. The biocompatible light‐curing resin was purchased from Boston Micro Fabrication Co., Ltd., China. Glucose powder for oral administration was obtained from Chongqing Heping Pharmaceutical Co., Ltd., China. NovolinInsulin penfill was obtained from Novo Nordisk A/S, China. The blood glucose test strips were purchased from Changsha Sinocare Inc., China. Compound dexamethasone acetate cream was purchased from Guangzhou BaiYunShan Pharmaceutical Holdings Co., Ltd., China. The lateral flow test strips for glucocorticoid detection were obtained from Shenzhen Finder Biotech Co., Ltd., China. BALB/c mice and NZ rabbits were purchased from Guangdong Medical Laboratory Animal Center, China. Deionized (D.I.) water was produced using the Millipore water purification system (18 MΩ, Milli‐Q, Millipore, U.S.A.).

### Design and Fabrication of the MAPs

The MAPs with different array numbers (5 × 5, 8 × 8, 9 × 9, 10 × 10) were fabricated using biocompatible resins on a micro‐nano 3D printer (nanoArchR S140, Boston Micro Fabrication Co., Ltd., China). The MAPs were designed with Cinema 4D software (Figure S15, Supporting Information), and the model files were exported to.STL file format. The model file was sliced by print preparation software (BMF 3D slicer, Boston Micro Fabrication Co., Ltd., China) into 420 template layers with 10‐µm thickness per layer. The template layers were classified into 5 sections according to the similarities between layers and the detailed printing parameters are shown in Table S1 (Supporting Information). After the 3D printing process was completed, the postprocessing steps were carried out as follows: First, a scraper carefully to take out the microneedle patch from the printing platform followed by washing it in the absolute ethanol bath for 20 min is used. During washing, the ethanol solution was agitated using a magnetic stirrer. Then, a syringe was used to rinse the chamber and hollow channels of MAPs with ethanol and deionized water, respectively, three times to remove the potential resin residues, followed by drying at room temperature. Finally, the MAP was post‐cured for another 20 min under a conventional UV box (λ = 405 nm) for full solidification, as schematically illustrated in Figure S2B, Supporting Information.

### Characterizations of the MAPs

Optical images of MAPs were captured by a smartphone (iPhone 12). The microstructure characterizations of MAPs were imaged using high‐resolution scanning electron microscopy (HRSEM, Thermo APREO S) with an accelerating voltage of 5 kV. Before SEM imaging, all samples were sputtered with a 10 nm gold coating (Leica Ltd, EM UC6). The force‐displacement tests of the MAPs were carried out by placing the MAPs with the microneedles upward on the stainless‐steel test platform of a universal mechanical tester (MTS CMT6103, China). For the compressive mechanical property characterization, the compressive stress (ó) – strain (ε) curve was obtained with the same universal mechanical tester. The constant speed of the load cell was 10 mm min^−1^. The slope of the stress‐strain curve during the elastic deformation was used as a measure of the compressive modulus and was calculated by $E\;=\frac{\overset{´}{o}}{\varepsilon }\;$. The mean compressive modulus was measured from different samples (*n* = 3).

### Assembly of the VT‐Integrated MAP Device

The device comprises three components: an MAP, a hose with an injection needle at one end, and a VT. When in use, the hose was first linked to the port of the MAP, and after inserting the MAP into the skin, the injection needle of the hose was inserted into the VT to form the whole extraction device.

### MAP Piercing Tests And H&E Staining

All animal experiments were approved by the Institutional Animal Care and Use Committee of Shenzhen University Medical School (Protocol no. IACUC SYXK2022‐0302) and procedures for animals were performed in accordance with the relevant guidelines and regulations. BALB/c male mice (10‐week‐old, ≈25–30 g) were anesthetized with pentobarbital sodium (50 mg kg^−1^ body weight, IP). Then, the dorsal hair of the anesthetized mouse was shaved. For the in vivo piercing tests, the MAP was inserted into the depilated back skin. The photos of the micropores formed in the mice's skin by the inserted MAP were captured by an optical microscope. For characterization of the skin recovery, the micropore images of the mice's skin were captured at 0, 5, 15, and 30 min after the penetration of the MAP. To investigate the influence of high vacuum on skin tissue, 10 kPa was provided for 5 min by a commercial negative pressure generator. The mice skin tissue specimen was fixed in 4.0% paraformaldehyde and embedded in paraffin for sectioning. The skin slides in 5‐µm thickness were stained by H&E staining for histological analysis. A pathological slice taken on a scanner (LEICA‐Aperio CS2) confirmed the successful penetration of the MAP.

### ISF Extraction using the VT‐Integrated MAP Device from Agarose Hydrogel Skin Model

First, the dry weight of the MAP device was recorded. Then, the MAP was pressed into the 1.5% (w/v) agarose hydrogel by thumb force followed by connecting the VT to generate negative pressure to extract the liquid from the hydrogel. 5 min later, the MAP was removed from the skin model. The wet mass of the MAP device was measured and gave the mass difference. In this way, the ISF extraction abilities of the device were evaluated. The extracted volume was calculated as the Equation (1):

(1) $$V\;=\frac{W_{w}-W_{d}}{\rho }\;$$

*W_d_* and *W_w_* are the dry and wet weight of the MAP device, respectively. The mean extraction volume was measured from *n* = 5 different samples.

### In Vivo ISF Extraction using the VT‐Integrated MAP Device from a Live NZ Rabbit Ear

Female NZ white rabbits (3‐month‐old, ≈1.5 kg) were anesthetized with 3% pentobarbital sodium (1 mL kg^−1^ body weight, IV). The ear hair of the anesthetized rabbits was shaved. After the dry weight of the MAP device was recorded, the MAPs were pressed into the rabbit ear skin by thumb force. Then, the injection needle end of the hose was inserted into the VT. Five minutes later, the MAP was removed and the wet mass of the device was recorded. The extracted volume in the MAP device and in the hose were calculated according to Equation 1 as mentioned above. The mean extraction volume was measured from *n* = 5 different samples.

### In Vivo Detection of ISF GC using the VT‐Integrated and LFTS‐Loaded MAP Device

The LFTSs for GC detection were purchased from Shenzhen Finder Biotech Co., Ltd (China). A piece of the LFTS was cut into a size of 1.5 × 20 mm for insertion into the hose to form the VT‐integrated and LFTS‐loaded MAP device. Female NZ white rabbits (3‐month‐old, ≈1.5 kg) were anesthetized with 3% pentobarbital sodium (1 mL kg^−1^ body weight, IV). The ear hair of the anesthetized rabbits was shaved. Compound dexamethasone acetate cream was dabbed onto the rabbit ear skin and left to allow the skin to fully absorb the cream. 30 min later after applying the cream, the MAP device was pressed onto the rabbit ear adjacent to the cream‐applied area to avoid the inference of the potential cream residue. Then, the VT was connected to the MAP patch to start the ISF extraction and GC detection. The results of ISF analysis were indicated visually by the test‐line (T‐line) and control line (C‐line).

### Preparation of the VT‐Integrated Chemiluminescent MAP Device

The nitrocellulose papers were first pretreated with 0.2% (w/v) chitosan solution and dried in an oven at 50 °C. A piece of the dried nitrocellulose paper was pre‐cut to an oblong shape with 10 mm length and 1.5 mm width. To prepare the chemiluminescent reagent solution, 1.0 mg mL^−1^ HRP and 100 U mL^−1^ LOx were dissolved in D.I. water. Then, 150 µL of the enzyme mixture was added into 2 mL of 2.0 mM luminol solution (pH 12.8, carbonate buffer solution). A 10 µL of the as‐prepared chemiluminescent reagent solution was pipetted on the chitosan‐treated paper, followed by drying in air, and then the resultant paper was inserted into the hose for use.

### Design and Fabrication of the 3D‐Printed Dark Box for CL Imaging

The lab‐made dark box was designed using 3D design software (Solidworks 3D CAD) and produced on a 3D printer (Farsoon HT403P, Farsoon Technologies) with black ABS polymers. Socket cap screws and copper nuts were used to connect the separate components. The dark box (13 × 6 × 6 cm) contained a liftable plate (5 × 5 cm) with a 0.5 cm‐depth groove for sample placement. The sample height could be adjusted with a knob on the outside of the box to make a clear focus for the snapshot. The smartphone adapter could be adjusted with 2 knobs on one side of the adaptor to fit the smartphone. After the smartphone was fixed and the sliding gate was closed, the dark box provided a suitable environment for CL signal snapping.

### In Vitro and in Vivo Detection of ISF Lactate using the VT‐Integrated Chemiluminescent MAP Device

A series of lactate standard solutions (0.5–30 mM) were prepared before testing. Then, 5 µL of standard solutions were introduced into different hoses to soak the nitrocellulose paper for chemiluminescent reactions. The photos of the hoses were captured by a dark‐box‐supported smartphone with all hoses placed at the same height and a suitable photography app (Slow Shutter, 5.7 Cogitap Software) to set the 30‐s exposure time for CL imaging. The intensities of light spots on the captured photographs were analyzed quantitatively using ImageJ software. Then, the relative light units of the CL signals were used to plot the calibration curve of the lactate concentrations. For blind testing, the eight different lactate concentrations as blind samples were tested with the chemiluminescent papers in the hose, and the results were verified by using a commercial CL analysis instrument.

For in vivo analysis, female NZ white rabbits (3‐month‐old, ≈1.5 kg) were anesthetized with 3% pentobarbital sodium (1 mL kg^−1^, IV). The ear hair of the anesthetized rabbit was shaved. Afterward, the VT‐integrated MAP device was used to extract the ISF and indicate the ISF lactate level.

### Preparation of the VT‐Integrated Colorimetric MAP Device

The nitrocellulose papers were first pretreated with 0.2% (w/v) chitosan solution and dried in an oven at 50 °C. A piece of the dried nitrocellulose paper was pre‐cut to an oblong shape with 10 mm length and 1.5 mm width. To prepare the glucose colorimetric papers, 10 mg of GOx, 0.3 mg of HRP, and 8 mM 4‐AAP were added into 1 mL of 0.1 M PBS buffer solution (pH 6.0). A 10 µL of the as‐prepared colorimetric reagent solution was pipetted on the chitosan‐treated paper, followed by drying in air, and then the resultant paper was inserted into the hose for use. Prior to use, the hoses were stored at 4 °C. To prepare the pH colorimetric papers, 5 µL of an ethanol solution containing 0.36 mM bromocresol green, 0.46 mM bromocresol red violet, and 0.08 mM bromothymol blue was dropped on a piece of bare nitrocellulose paper to according to the aforementioned procedures.

### In Vitro and in Vivo Detection of ISF Glucose and pH using the VT‐Integrated Colorimetric MAP Device

A series of glucose standard solutions (0–40 mM) and pH standard solutions (pH 3.0–9.0) were prepared before testing. Then, 5 µL of standard solutions were introduced into different hoses to soak the nitrocellulose paper for colorimetric reactions. The photos of the hoses were captured by a smartphone. To avoid the influence of outdoor light and minimize the interferences of the stray light, all photos were captured within a lab‐made device equipped with the same smartphone, the same light source, the same sample‐smartphone distance, and the same sample orientation. The color changes were processed on a lab‐designed smartphone app based on the code of OpenCV lib (Python, Pycharm Community Edition 2023.1) to output automatically the red (R), green (G), and blue (B) values. Then, the R values were used to plot the calibration curve of the glucose concentrations and pH values. The bare nitrocellulose paper was chosen as the reference material. For blind testing, the eight different glucose and pH concentrations as blind samples were tested with the chemiluminescent papers in the hose, and the results were verified by using commercial analysis instruments.

Female NZ white rabbits (3‐month‐old, ≈1.5 kg) were anesthetized with 3% pentobarbital sodium (1 mL kg^−1^, IV). The ear hair of the anesthetized rabbit was shaved. Afterward, the VT‐integrated MAP device was used to extract the ISF and show the colorimetric results of ISF glucose and pH. The color changes were processed on a lab‐designed smartphone app to output automatically the R values and the corresponding analyte concentrations. The extraction position on the rabbit ear skin was the same. There was an interval of 20 min between every trial of detection, meanwhile, gently effleurage was given to improve the recovery of the testing ear skin. For comparison, the blood glucose and pH levels in the rabbit ear were also analyzed using a commercial glucometer and a pH meter, respectively.

### In Vivo Glucose Level Monitoring using the VT‐Integrated Colorimetric MAP Device

Oral glucose intake and insulin intramuscular injection were carried out in a one‐day experiment. The rabbits were not fed for 12 h before the experiment. After depilation of the rabbits followed by the first penetration of the skin with the MAP, to simulate the glucose variations in daily physiologic activities of rabbits, at the time point of the 100th min, the rabbit was fed with a sugar drink containing 3 g kg^−1^ glucose and another 100 min later, 1 U kg^−1^ insulin was administered intramuscularly. The VT‐integrated MAP device was used to extract the ISF and indicate the glucose concentrations at every point in time. Meanwhile, a commercial glucometer was used to verify the ISF glucose level. For comparison, the rabbit ear vein blood glucose level was also detected by the glucometer.

### Statistical Analysis

The data were presented as the mean ± s.e.m., as indicated. Statistical analysis was carried out with Origin software (Origin 2022, Origin Software). For group comparison, one‐way ANOVA was used. Statistical significance was denoted by ^*^ *p* < 0.05, ^**^ *p* < 0.01, and ^***^ *p *< 0.001.

## Conflict of Interest

The authors declare no conflict of interest.

## Author Contributions

Y.T.X. participated in the conception of the original project, designed and performed experiments, analyzed data, and participated in the figure design and writing and revising of the manuscript. J.H.H. and C.J.Z. performed microneedle fabrications. W.Q.H. contributed to the app design and development. T.I. reviewed and edited the manuscript. L.S. conceived the original project, designed experiments, wrote, and revised the manuscript. X.J.Z. participated in the discussion of the research.

## Supporting information

Supporting Information

Supporting Information

Supporting Information

## Data Availability

The data that support the findings of this study are available from the corresponding author upon reasonable request.
