# Supplementary material for: Enhanced Interstitial Fluid Extraction and Rapid Analysis via Vacuum Tube‐Integrated Microneedle Array Device
Source: Adv Sci (Weinh). 2024 Mar 19;11(21):2308716. doi: 10.1002/advs.202308716 (PMC11151006; doi:10.1002/advs.202308716)
Supplement: Supplementary file 1 — Supporting Information [file ADVS-11-2308716-s002.pdf]

ADVANCED SCIENCE
Open Access

Supporting Information

for *Adv. Sci.*, DOI 10.1002/advs.202308716

Enhanced Interstitial Fluid Extraction and Rapid Analysis via Vacuum Tube-Integrated Microneedle Array Device

Yuanting Xie, Jinhua He, Wenqing He, Tayyaba Iftikhar, Chuangjie Zhang, Lei Su and Xueji Zhang**

Supporting Information

Enhanced Interstitial Fluid Extraction and Rapid Analysis via Vacuum Tube-Integrated Microneedle Array Device

Yuanting Xie, Jinhua He, Wenqing He, Tayyaba Iftikhar, Chuangjie Zhang, Lei Su [*], *and Xueji Zhang* [*]

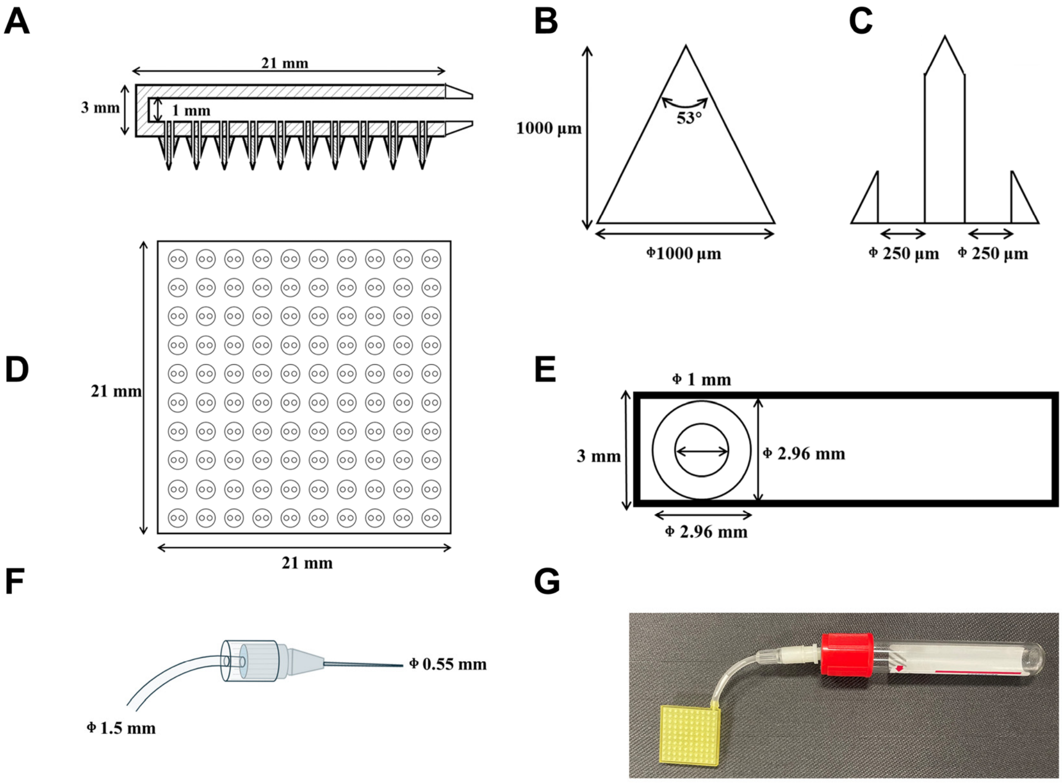

Figure S1. **The detailed geometry of the microneedle-based ISF extraction device.** (A) The cross-section view of the MAP device with chamber. (B) and (C) The parametric design of single needle tip. (D) The top view of the MAP design. (E) The parametric design of the outlet port. (F) The detailed geometry of the hose. (G) The overall image of the vacuum tube-integrated MAP device.

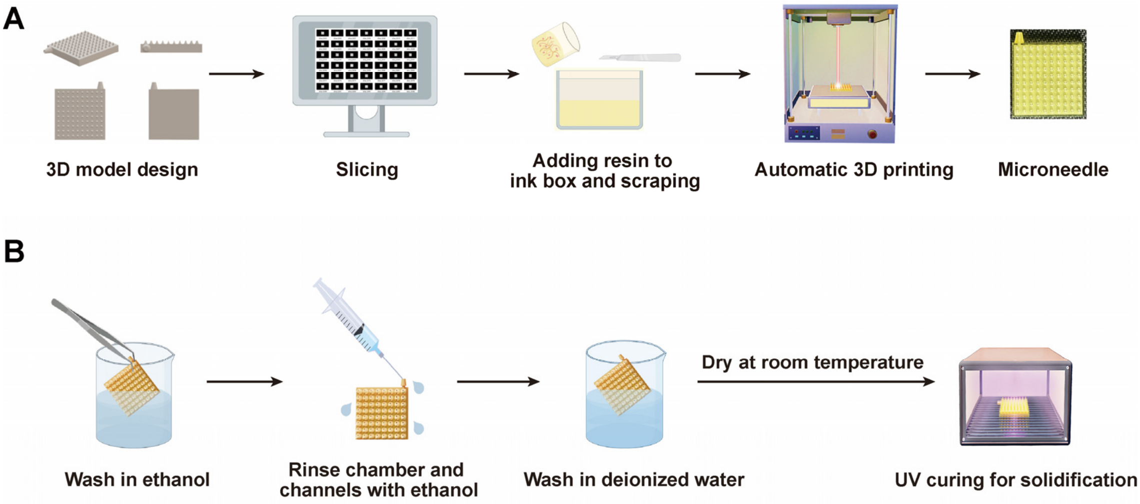

Figure S2. Schematic illustration of the MAP-based device fabrication (A) and postprocessing steps (B).

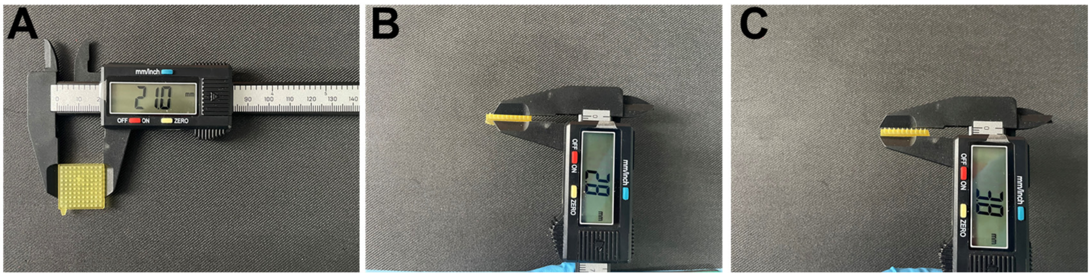

Figure S3. Views of a 10×10 MAP and the size of 10×10 MAP measured by a vernier caliper. (A) The length of the 10×10 MAP. (B) The width of the 10×10 MAP chamber. (C) The height of the 10×10 MAP (miconeedle tips involved).

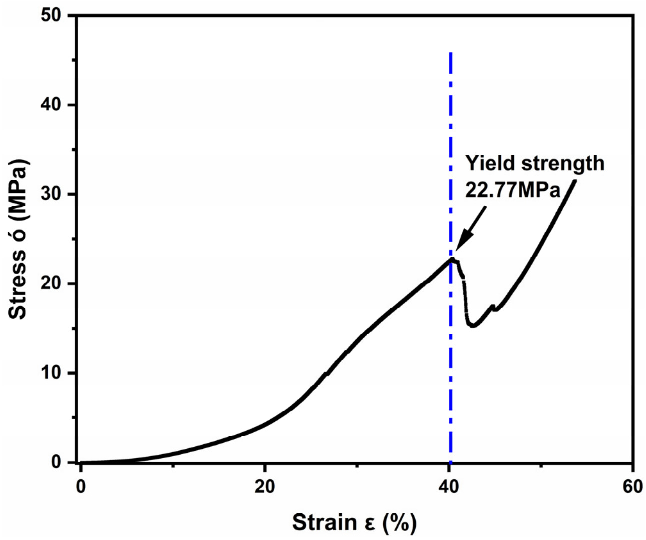

Figure S4. Stress-strain diagram of the MAP. The arrow indicates the yield strength of the MAP.

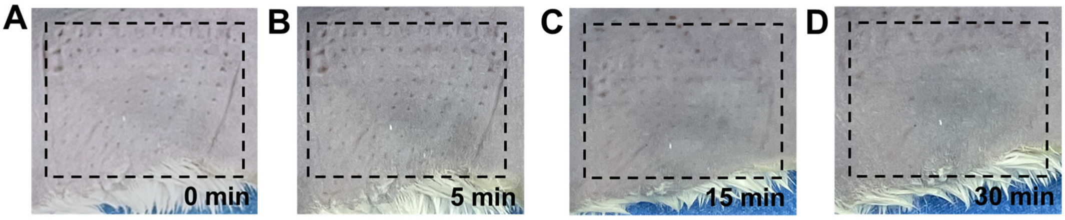

Figure S5. The skin recovery after the application of MAP penetration of the mice dorsal skin at the time points of 0 min (A), 5 min (B), 15 min (C), and 30 min (D), respectively.

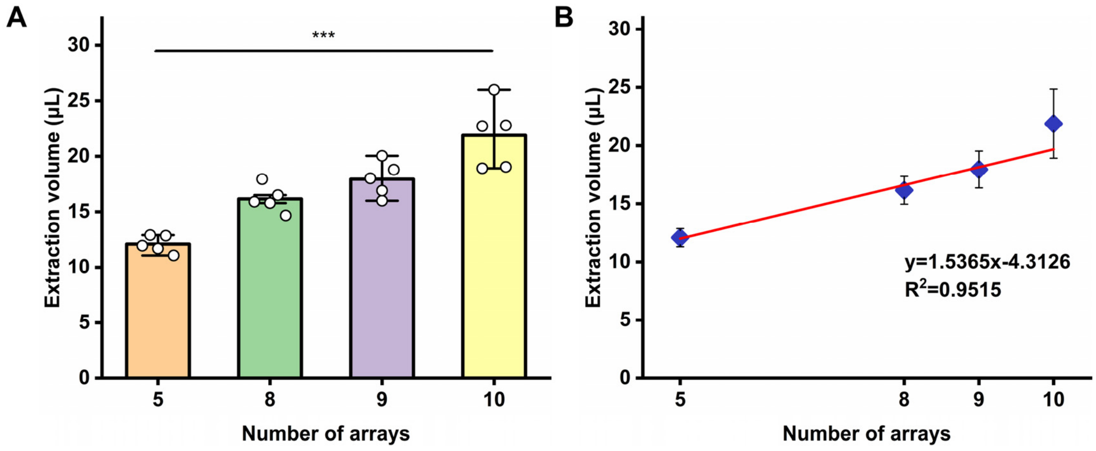

Figure S6. (A) The extraction performance of the VT-coupled MAP device with different microneedle array numbers (5 × 5, 8 × 8, 9 × 9, 10 × 10) within 5 minutes. (B) Correlation between the amount of collected ISF from the agarose skin models and the number of MN arrays. Data are presented as mean±s.e.m.. P-values were calculated using one-way ANOVA. *p<0.05, **p<0.01, and ***p<0.001.

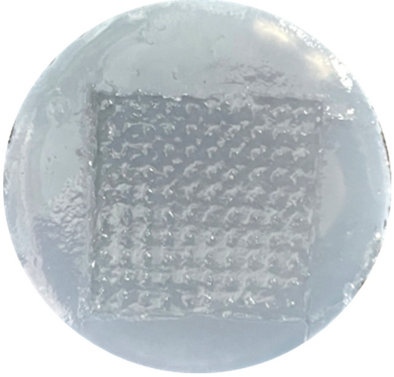

Figure S7. The photo of the agarose gel after the removal of the MAP under a 5-min application of a 10 kPa negative pressure.

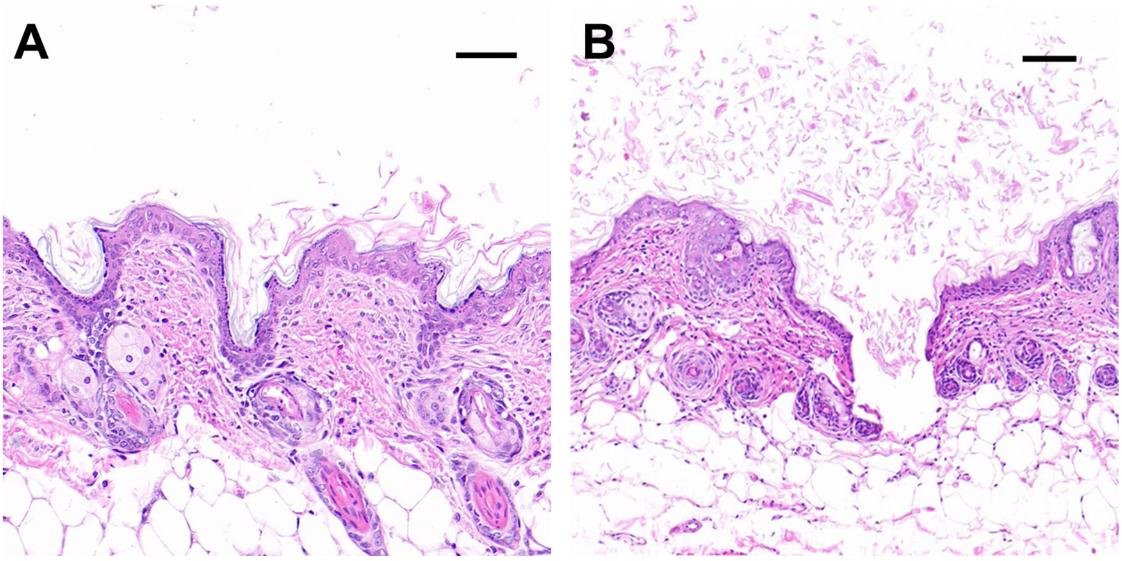

Figure S8. H&E-staining of the mouse skin pierced by the MAP with different vacuum degrees. (A) H&E-staining of the mouse skin which was applied with the VT-coupled MAP device. (B) H&E-staining of the mouse skin which was applied with the MAP under a 10 kPa vacuum. Scale bar: 50 μm.

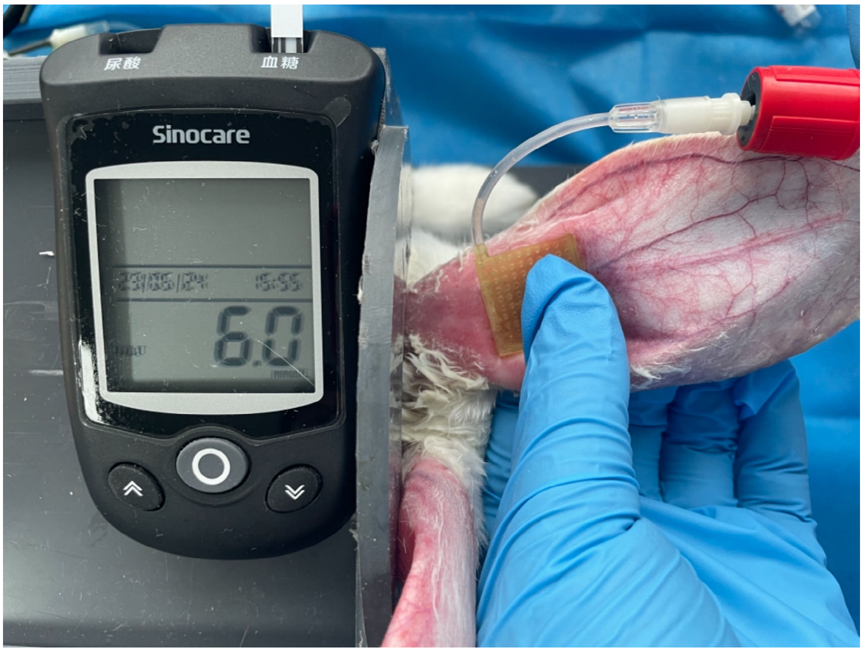

Figure S9. In vivo detection of the glucose in the ISF extracted using the VT-coupled MAP device from the rabbit ear skin with a glucometer.

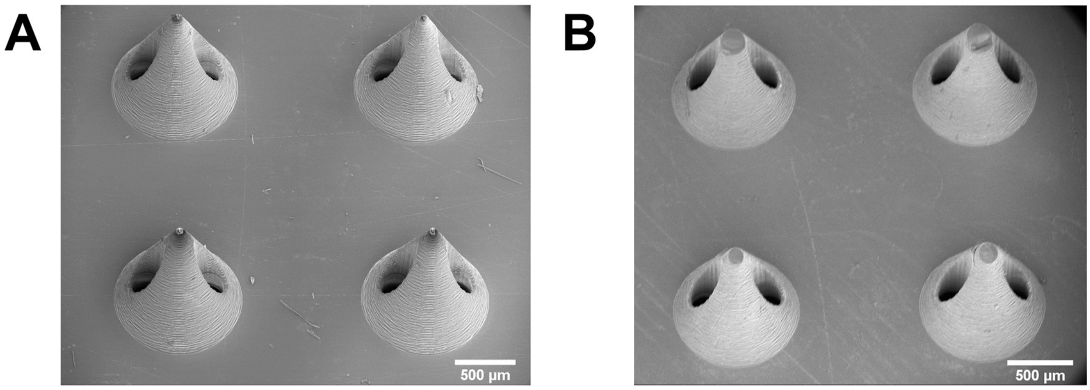

Figure S10. SEM images of microneedles before (A) and after compression test (B).

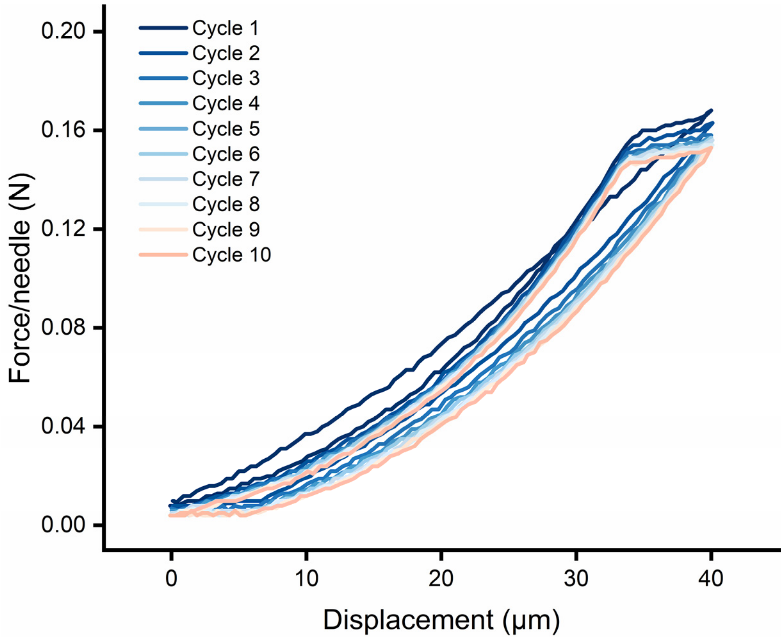

Figure S11. The mechanical compressive cycle test of the MAP for 10 times.

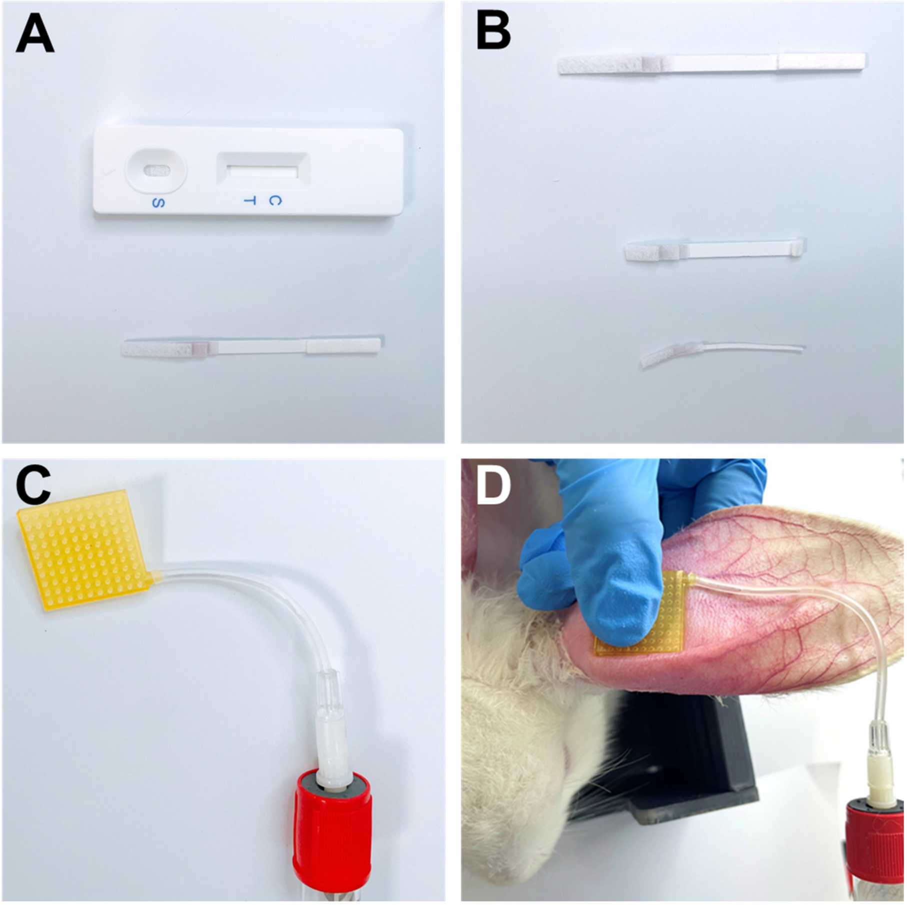

Figure S12. The VT-coupled and LFTS-functionalized MAP device for rapid extraction and lateral flow analysis of ISF. (A) The commercial GC lateral flow test kit (top) and the LFTS taken out from the kit (bottom). (B) A piece of LFTS with the size of 1.5×20 mm (bottom) tailored from a piece of factory-fresh LFTS (top). (C) The photo of the VT-coupled and LFTS-functionalized MAP device. (D) The photo of the VT-coupled MAP device for in vivo detection of GC in the rabbit ear skin.

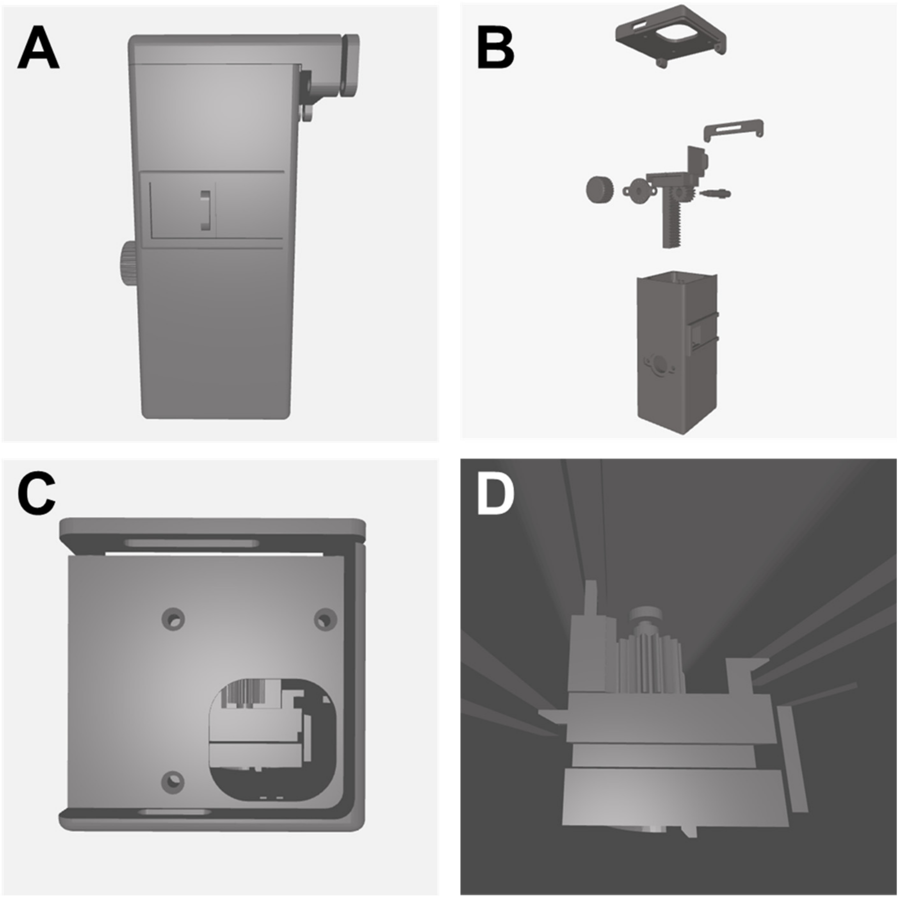

Figure S13. Design and 3D modeling of the lab-made dark box on the Cinema4D modeling software. (A) Side view. (B) Diagram of assembly units. (C) Vertical view. (D) Internal vertical view.

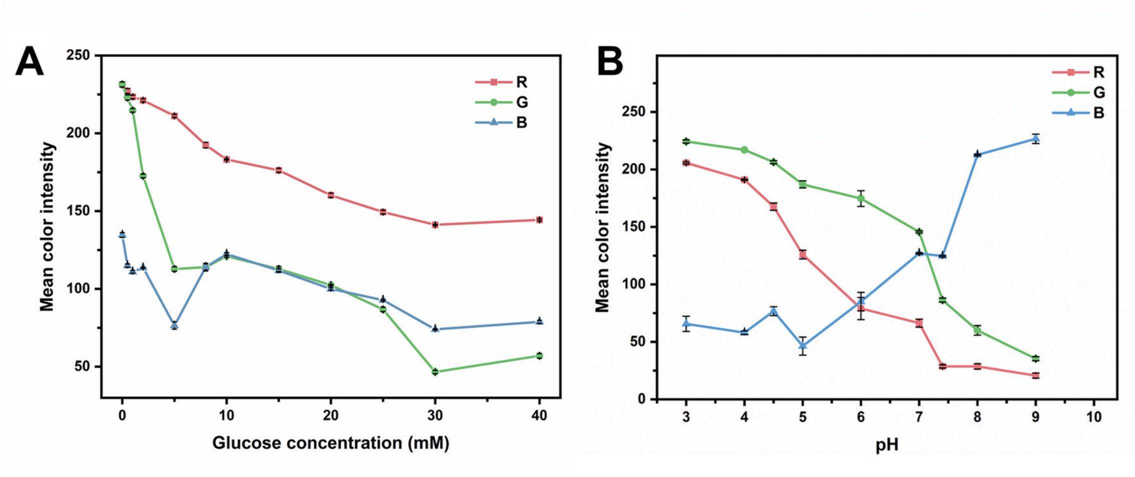

Figure S14. Colorimetric analysis of the ISF glucose and pH based on the data from the sensing hose of the VT-coupled MAP device. (A) Plot of R, G, B values vs glucose concentrations. (B) Plot of R, G, B values vs pH values.

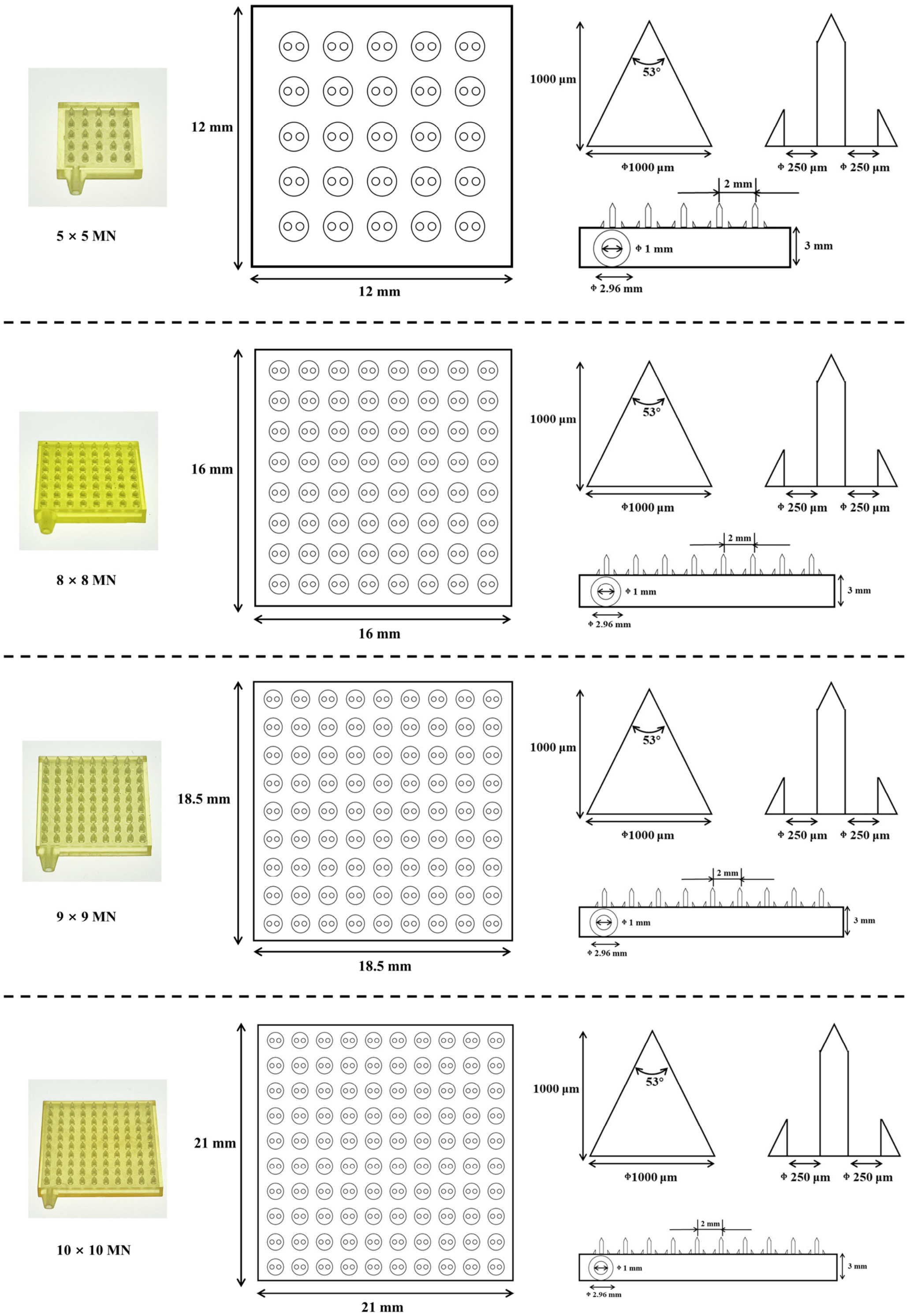

Figure S15. Designs and dimensions of the MAPs used for the experiments. (A) Images and 2D drawings of the 5 × 5 MAP. (B) Images and 2D drawings of the 8 × 8 MAP. (C) Images and 2D drawings of the 9 × 9 MAP. (D) Images and 2D drawings of the 10 × 10 MAP.

Table S1. Parameter setting for micron printing process.

Section No.	Template layer	Exposure time (UV) [second]	Exposure intensity (UV) [mW/cm^2]	Frequency of scraper movement [a)]
1	1 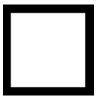	5.00	56	0
2	99 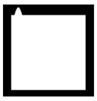	4.00	47	4
3	100 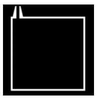	4.00	47	4
4	100 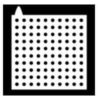	3.00	39	5
5	120 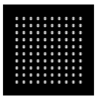	3.00	39	2

[a)] Scraping the liquid resin surface after printing for certain layers to avoid bubbles during precision printing.

Table S2. Summary table of recent studies on ISF extraction techniques.

Type of MN	MN parameter	Object Model	Flow Mechanism	ISF collection rate	Postprocessing after extraction	Ref
Solid MNs	700-1500 μm, 7 MNs	Anesthetized rat	Hole puncturing	1-10 μL in 10 min	/	[1]
Hollow MNs	1500 μm, 5 MNs	In vivo rat	Capillary force	16 μL in 1-2 h	/	[2]
	250 μm, 5 MNs	Human	Pressure/suction	0.01-0.03 μL in 20 min	/	[3]
	1500 μm, 81 MNs	In vivo rat	Pressure	17.03 ± 1.40 μL h^{-1}	/	[4]
	250 μm, 5 MNs	Human	Pressure	2.3 ± 2.6 μL in 20 min	/	[5]
Hydrogel MNs	900 μm, 100 MNs	In vivo mouse	Osmosis and swelling	3.82 μL in 3 min	Centrifugation	[6]
	800 μm, 100 MNs	In vivo rat	Swelling	6.3 ± 0.6 μL in 45 min	/	[7]
	600 μm, 121 MNs	In vivo rat	Swelling	2.67 mg in 30 min	Centrifugation	[8]
	600 μm, 100 MNs	In vivo rat	Swelling	0.84 ± 0.24 mg h^{-1}	/	[9]
	1266 μm, 100 MNs	In vivo rabbit	Swelling	1.25 ± 0.37 μL in 10 min	Heat and centrifugation	[10]
Porous MNs	900 μm, 9 MNs	Agarose gel	Capillary force	0.667 μL/min (estimated flow rate)	Manual compression	[11]
	37 MNs	Ex vivo porcine skin	Electroosm-otic flow	40 μL cm^{-2} h^{-1}	/	[12]
Sponge Forming MNs	680 μm, 144 MNs	In vivo rat	Absorption and swelling	1.6 μL in 1 min	Centrifugation	[13]
This work	1000 μm, 100 MNs	In vivo rabbit	Negative pressure	18.42 μL in 5 min	Directly extract and store	/

References

[1] M. C. Ping M. Wang, and Mark R. Prausnitz., *Diabetes Technology & Therapeutics* **2005**, 7, 131.

[2] P. R. Miller, R. M. Taylor, B. Q. Tran, G. Boyd, T. Glaros, V. H. Chavez, R. Krishnakumar, A. Sinha, K. Poorey, K. P. Williams, S. S. Branda, J. T. Baca, R. Polsky, *Communications Biology* **2018**, 1, 173.

[3] P. P. Samant, M. R. Prausnitz, *Proceedings of the National Academy of Sciences* **2018**, 115, 4583.

[4] T. Abbasiasl, F. Mirlou, H. Mirzajani, M. J. Bathaei, E. Istif, N. Shomalizadeh, R. E. Cebecioğlu, E. E. Özkahraman, U. C. Yener, L. Beker, *Advanced Materials* **2024**, 36, 2304704.

[5] P. P. Samant, M. M. Niedzwiecki, N. Raviele, V. Tran, J. Mena-Lapaix, D. I. Walker, E. I. Felner, D. P. Jones, G. W. Miller, M. R. Prausnitz, *Science Translational Medicine* **2020**, 12, eaaw0285.

[6] M. Zheng, Z. Wang, H. Chang, L. Wang, S. W. T. Chew, D. C. S. Lio, M. Cui, L. Liu, B. C. K. Tee, C. Xu, *Advanced Healthcare Materials* **2020**, 9, 1901683.

[7] E. Laszlo, G. De Crescenzo, A. Nieto-Argüello, X. Banquy, D. Brambilla, *Advanced Functional Materials* **2021**, 31, 2106061.

[8] J. Zhu, X. Zhou, H.-J. Kim, M. Qu, X. Jiang, K. Lee, L. Ren, Q. Wu, C. Wang, X. Zhu, P. Tebon, S. Zhang, J. Lee, N. Ashammakhi, S. Ahadian, M. R. Dokmeci, Z. Gu, W. Sun, A. Khademhosseini, *Small* **2020**, 16, 1905910.

[9] A. V. Romanyuk, V. N. Zvezdin, P. Samant, M. I. Grenader, M. Zemlyanova, M. R. Prausnitz, *Analytical Chemistry* **2014**, 86, 10520.

[10] R. He, Y. Niu, Z. Li, A. Li, H. Yang, F. Xu, F. Li, *Advanced Healthcare Materials* **2020**, 9, 1901201.

[11] K. Takeuchi, N. Takama, B. Kim, K. Sharma, O. Paul, P. Ruther, *Biomedical Microdevices* **2019**, 21, 28.

[12] S. Kusama, K. Sato, Y. Matsui, N. Kimura, H. Abe, S. Yoshida, M. Nishizawa, *Nature Communications* **2021**, 12, 658.

[13] J. Chen, M. Wang, Y. Ye, Z. Yang, Z. Ruan, N. Jin, *Biomedical Microdevices* **2019**, 21, 63.
